# Supplementary material for: A Hypovirulence-Associated Partitivirus and Re-Examination of Horizontal Gene Transfer Between Partitiviruses and Cellular Organisms
Source: Int J Mol Sci. 2025 Apr 18;26(8):3853. doi: 10.3390/ijms26083853 (PMC12027680; doi:10.3390/ijms26083853)
Supplement: Supplementary file 1 [file ijms-26-03853-s001.zip › Table S1-20241006.pdf]

Table S1: Primers used in this study

Primer	Sequence	bp	Function
MRF1567	CCCGAAGAATGTGAATCCG	19	detect for SsAPV1 RdRP
MRR1567	AGGTAGGGAAGTGCGACAAAT	21	
MCF1476	ATGTCCTCCAACGCCACC	18	detect for SsAPV1 CP
MCR1476	GACACGGGACTTGACCATGC	20	
5APV1RS	ACGATTGGGGTTCTAGGCTG	20	For 3′ terminal-sequence cloning of SsAPV1 RdRp
5APV1RL	TCGTAAGCAAGAGCCGATGA	20	
3APV1RS	GTCAGCTGCTAACCCCAAGA	20	For 5′ terminal sequence cloning of SsAPV1 RdRp
3APV1RL	AACAAGCATTCGTTCTCGGC	20	
5APV1CS	CGTTCAACGCATTTCCGTCT	20	For 3′ terminal-sequence cloning of SsAPV1 CP
5APV1CL	GAAACTTAGACACGCCGCTG	20	
3APV1CS	TGTCGGGGATGGTCATCTTG	20	For 5′ terminal sequence cloning of SsAPV1 CP
3APV1CL	TCCAGCGCTTAGTATCAGCC	20	
PC2	CCGAATTCCCGGGATCC	17	For nested PCR
contig78F	TGCCCAAAGAGTGCTGAATG	20	detect for SsOLV20
contig78R	AGGTGTTGAGGTGGACGAGAA	21	
contig244F	GGGTCCCTTCACTGGGTTATC	21	detect for MpMV1
contig244R	TTACCTTTCCTGCCGCCTC	19	
contig84F	GGCTGGAACTTGCGTCATC	19	detect for SsOLV7
contig84R	CAATCGTCTTGTCCTTGAATAGAGT	25	

contig282F	CGCTGACAGAGGTGCCATT	19	detect for SsMV14
contig282R	AACGAGTTACCGCTTTCCATT	21	
contig177F	GCTCCGAGGGATTGAAGGT	19	detect for SsMV6
contig177R	CGACTTGAGTGGGAAGGGTAT	21	
contig69F	TATCAGGATTCATACCGAGGCA	22	detect for SsMV9
contig69R	CACCGACAAAGGAAAGAAGGAG	22	
contig166F	AGACTGTCGGTCTAAACGGATGT	23	detect for SsOLV12
contig166R	CACTCGGATTTCTTCGCTCAA	21	
contig526F	AAGTGCTGTTGTTGCCTGACC	21	detect for SsElV1
contig526R	TGGGACTATGAAATGGCTGAA	21	
R5UTRF	CAAATCTTTCGGATCCCTCGAC	22	For nested PCR of SsAPV1 RdRP; detect for DNA segment of SsAPV1 RdRP
R5SR309	GAGTTGAGCTTGTCGATGTCCT	22	
R5LR568	GCGTCGGTGTTTGAAATCAGTG	22	
CP5UTRF	TCAAATCTTTCGGAATCCTCCGT	23	For nested PCR of SsAPV1 CP; detect for DNA segment of SsAPV1 CP
CP5SR295	ACTCTTCTTCATATCGGGAGCG	22	
CP5LR507	GGGTGATAATCAGGACAGTAGCG	23	
PC3-T7	GGATCCCGGGAATTCGGTAATACG ACTCACTATATTTTTATAGTGAGTCGTATTA	55	As adaptor primer for viral terminal sequence cloning
