# Supplementary material for: A Hypovirulence-Associated Partitivirus and Re-Examination of Horizontal Gene Transfer Between Partitiviruses and Cellular Organisms
Source: Int J Mol Sci. 2025 Apr 18;26(8):3853. doi: 10.3390/ijms26083853 (PMC12027680; doi:10.3390/ijms26083853)
Supplement: Supplementary file 1 [file ijms-26-03853-s001.zip › Table S2-20241006.pdf]

**Table S2: Viruses selected for phylogenetic analysis in this study**

| Viruses | Aberr. | RdRP | CP | Genus | Group |
|---|---|---|---|---|---|
| Medicago sativa alphapartitivirus 2 | MsAPV2 | QBC36014.1 | QBC36015.1 | *Alphapartitivirus* | plant virus |
| Medicago sativa alphapartitivirus 1 | MsAPV1 | YP_009551597.1 | YP_009551596.1 | *Alphapartitivirus* | plant virus |
| Pear alphapartitivirus | PpPV2 | BBA66577.1 | BBA66578.1 | *Alphapartitivirus* | plant virus |
| Rhizoctonia oryzae-sativae partitivirus 1 | RosPV1 | AYV61425.1 | AYV61426.1 | *Alphapartitivirus* | fungal virus |
| Rose partitivirus | RoPV-PB | ANQ45203.1 | ANQ45204.1 | *Alphapartitivirus* | plant virus |
| Bipolaris maydis partitivirus 1 | BmPV1 | YP_009362092.1 | YP_009362099.1 | *Alphapartitivirus* | fungal virus |
| Arabidopsis halleri partitivirus 1 | AhPV1 | YP_009273018.1 | YP_009273019.1 | *Alphapartitivirus* | plant virus |
| Vicia faba partitivirus 1 | VfPV1 | ABJ99996.1 | | *Alphapartitivirus* | plant virus |
| Raphanus sativus partitivirus 1 | RsPV1 | ALT00589.1 | | *Alphapartitivirus* | plant virus |
| Rosellinia necatrix partitivirus 2 | RnPV2 | YP_007419077.1 | YP_007419078.1 | *Alphapartitivirus* | fungal virus |
| Flammulina velutipes browning virus | FvBV | YP_009508048.1 | YP_009508047.1 | *Alphapartitivirus* | fungal virus |
| Heterobasidion partitivirus 1 | HetPV1 | YP_009508049.1 | YP_009508050.1 | *Alphapartitivirus* | fungal virus |
| Botrytis cinerea partitivirus 2 | BcPV2 | AXI91519.1 | AXI91520.1 | *Alphapartitivirus* | fungal virus |
| Sclerotinia sclerotiorum partitivirus S | SsPV-S | YP_003082248.1 | YP_003082249.1 | *Alphapartitivirus* | fungal virus |
| White clover cryptic virus 1 | WCCV1 | YP_086754.1 | YP_086755.1 | *Alphapartitivirus* | plant virus |
| Dill cryptic virus 1 | DCV1 | YP_008719880.1 | YP_008719881.1 | *Alphapartitivirus* | plant virus |
| Beet cryptic virus 1 | BCV1 | YP_002308574.1 | YP_002308575.1 | *Alphapartitivirus* | plant virus |
| Carrot cryptic virus | CarCV | YP_009508045.1 | YP_009508046.1 | *Alphapartitivirus* | plant virus |
| Sclerotinia sclerotiorum partitivirus 1 | SsPV1 | AFR78160.1 | AFR78159.1 | *Betapartitivirus* | fungal virus |

| | | | | | |
|---|---|---|---|---|---|
| Cannabis cryptic virus | CanCV | YP_009293586.1 | YP_009293580.1 | *Betapartitivirus* | plant virus |
| Crimson clover cryptic virus 2 | CCCV2 | YP_009508059.1 | YP_009508060.1 | *Betapartitivirus* | plant virus |
| Dill cryptic virus 2 | DCV2 | YP_007891054.1 | YP_007891055.1 | *Betapartitivirus* | plant virus |
| Hop trefoil cryptic virus 2 | HTCV2 | YP_007889825.1 | YP_007889826.1 | *Betapartitivirus* | plant virus |
| Primula malacoides virus 1 | PmV1 | YP_003104768.1 | YP_003104769.1 | *Betapartitivirus* | plant virus |
| Red clover cryptic virus 2 | RCCV2 | YP_007889823.1 | YP_007889824.1 | *Betapartitivirus* | plant virus |
| White clover cryptic virus 2 | WCCV2 | YP_007889821.1 | YP_007889822.1 | *Betapartitivirus* | plant virus |
| Atkinsonella hypoxylon virus | AhV | NP_604475.1 | NP_604476.1 | *Betapartitivirus* | fungal virus |
| Ceratocystis resinifera virus 1 | CrV1 | YP_001936016.1 | YP_001936015.1 | *Betapartitivirus* | fungal virus |
| Fusarium poae virus 1 | FpV1 | NP_624349.1 | NP_624348.1 | *Betapartitivirus* | fungal virus |
| Heterobasidion partitivirus 2 | HetPV2 | YP_009508061.1 | YP_009508062.1 | *Betapartitivirus* | fungal virus |
| Heterobasidion partitivirus 8 | HetPV8 | YP_009508063.1 | YP_009508064.1 | *Betapartitivirus* | fungal virus |
| Pleurotus ostreatus virus 1 | PoV1 | YP_227355.1 | YP_227354.1 | *Betapartitivirus* | fungal virus |
| Rhizoctonia solani virus 717 | RHsV717 | NP_620659.1 | NP_620660.1 | *Betapartitivirus* | fungal virus |
| Rosellinia necatrix partitivirus 1 | RnPV1 | YP_392480.1 | YP_392481.1 | *Betapartitivirus* | fungal virus |
| Heterobasidion partitivirus 7 | HetPV7 | YP_009408640.1 | YP_009408641.1 | *Betapartitivirus* | fungal virus |
| Aspergillus ochraceous virus | AoV | YP_009665972.1 | YP_009665973.1 | *Gammapartitivirus* | fungal virus |
| Discula destructiva virus 1 | DdV1 | NP_116716.1 | NP_116742.1 | *Gammapartitivirus* | fungal virus |
| Discula destructiva virus 2 | DdV2 | NP_620301.1 | NP_620302.1 | *Gammapartitivirus* | fungal virus |
| Fusarium solani virus 1 | FsV1 | NP_624350.1 | NP_624351.1 | *Gammapartitivirus* | fungal virus |
| Gremmeniella abietina RNA virus MS1 | GaRV-MS1 | NP_659027.1 | NP_659028.1 | *Gammapartitivirus* | fungal virus |

| | | | | | |
|---|---|---|---|---|---|
| Ophiostoma partitivirus 1 | OPV1 | YP_009508238.1 | YP_009508237.1 | *Gammapartitivirus* | fungal virus |
| Penicillium stoloniferum virus S | PsV-S | YP_052856.2 | YP_052857.1 | *Gammapartitivirus* | fungal virus |
| Aspergillus fumigatus partitivirus 1 | AfuPV1 | CAY25801.2 | CAZ61323.2 | *Gammapartitivirus* | fungal virus |
| Botryotinia fuckeliana partitivirus 1 | BfPV1 | YP_001686789.1 | YP_001686790.1 | *Gammapartitivirus* | fungal virus |
| Ustilaginoidea virens partitivirus 1 | UvPV1 | AGO04402.1 | AGO04403.1 | *Gammapartitivirus* | fungal virus |
| Verticillium dahliae partitivirus 1 | VdPV1 | YP_009164038.1 | YP_009164039.1 | *Gammapartitivirus* | fungal virus |
| Beet cryptic virus 2 | BCV2 | YP_009508068.1 | YP_009508067.1 | *Deltapartitivirus* | plant virus |
| Fig cryptic virus | FCV | YP_004429258.1 | YP_004429259.1 | *Deltapartitivirus* | plant virus |
| Pepper cryptic virus 1 | PepCV1 | YP_009466859.1 | YP_009466860.1 | *Deltapartitivirus* | plant virus |
| Pepper cryptic virus 2 | PepCV2 | YP_009351838.1 | YP_009351848.1 | *Deltapartitivirus* | plant virus |
| Fragaria chiloensis cryptic virus | FcCV | YP_001274391.1 | YP_001274392.1 | *Deltapartitivirus* | plant virus |
| Persimmon cryptic virus | PerCV | YP_006390091.1 | YP_006390090.1 | *Deltapartitivirus* | plant virus |
| Raphanus sativus cryptic virus 2 | RsCV2 | YP_001686783.1 | YP_001686784.1 | *Deltapartitivirus* | plant virus |
| Raphanus sativus cryptic virus 3 | RsCV3 | YP_002364401.1 | YP_002364402.1 | *Deltapartitivirus* | plant virus |
| Rose cryptic virus 1 | RoCV1 | YP_001686786.1 | YP_001686787.1 | *Deltapartitivirus* | plant virus |
| Cryptosporidium parvum virus 1 | CSpV1 | YP_009508065.1 | YP_009508066.1 | *Cryspovirus* | insect virus |
| Alternaria alternata partitivirus 1 | AaPV1 | APT70073.1 | APT70074.1 | *Zetapartitivirus* | fungal virus |
| Botryosphaeria dothidea partitivirus 1 | BdPV1 | AGZ84316.1 | AGZ84317.1 | *Zetapartitivirus* | fungal virus |
| Penicillium brasilianum partitivirus 1 | PbPV1 | AZT88608.1 | AZT88609.1 | *Zetapartitivirus* | fungal virus |
| Delitschia confertaspora partitivirus 1 | DcPV1 | AZT88584.1 | AZT88585.1 | *Zetapartitivirus* | fungal virus |
| Penicillium aurantiogriseum partiti-like virus 1 | PaPlV1 | YP_009182157.1 | ASY04022.1 | *Epsilonpartitivirus* | fungal virus |

| | | | | | |
|---|---|---|---|---|---|
| Colletotrichum eremochloae partitivirus 1 | CePV1 | AZT88590.1 | AZT88591.1 | *Epsilonpartitivirus* | fungal virus |
| Hubei partiti-like virus 11 | HplV11 | YP_009329875.1 | YP009329874.1 | *Epsilonpartitivirus* | insect virus |
| Rhizoctonia solani dsRNA virus 5 | RsRV5 | AVP26802.1 | AVP26801.1 | *Epsilonpartitivirus* | fungal virus |
| Saccharomyces cerevisiae partitivirus 1 | ScPV1 | WGJ63525.1 | WGJ63526.1 | *Unclassfied* | fungal virus |
| Saccharomyces cerevisiae partitivirus 2 | ScPV2 | WGJ63524.1 | WGJ63523.1 | *Unclassfied* | fungal virus |
| Saccharomyces cerevisiae partitivirus 3 | ScPV3 | WGJ63527.1 | WGJ63528.1 | *Unclassfied* | fungal virus |
| Leuven Partiti-like virus 5 | LeuPLV5 | QZZ63414.1 | | *Unclassfied* | insect virus |
| Hubei partiti-like virus 19 | HuPLV19 | APG78260.1 | | *Unclassfied* | insect virus |
| Atrato Partiti-like virus 6 | AtPLV6 | QHA33706.1 | | *Unclassfied* | insect virus |
| Enontekio partiti-like virus | EnPLV | UUV42340.1 | | *Unclassfied* | insect virus |
| Lestijarvi partiti-like virus | LesPLV | UUV42382.1 | | *Unclassfied* | insect virus |
| Vaasa partiti-like virus | VaPLV | UUV42391.1 | | *Unclassfied* | insect virus |
| Hubei partiti-like virus 22 | HuPLV22 | BBQ05104.1 | | *Unclassfied* | insect virus |
| Hameenlinna partiti-like virus | HatPLV | UUV42344.1 | | *Unclassfied* | insect virus |
| Kagbo partiti-like virus | KaPLV | UYE93839.1 | | *Unclassfied* | insect virus |
| Spodoptera exempta insect virus 1 | SeIV1 | QFR15908.1 | | *Unclassfied* | insect virus |
| Hattula partiti-like virus | HamPLV | UUV42351.1 | | *Unclassfied* | insect virus |
| Penicillium chrysogenum virus | PcV | YP_392482.1 | YP_392483.1 | *Chrysoviridae* | fungal virus |
