# Supplementary material for: A Hypovirulence-Associated Partitivirus and Re-Examination of Horizontal Gene Transfer Between Partitiviruses and Cellular Organisms
Source: Int J Mol Sci. 2025 Apr 18;26(8):3853. doi: 10.3390/ijms26083853 (PMC12027680; doi:10.3390/ijms26083853)
Supplement: Supplementary file 1 [file ijms-26-03853-s001.zip › Table S3-20241006.pdf]

Table S3: Partitivirus RdRP-like proteins in cellular organisms

Scientific Name	Accession	protein	Length	Group	Family	Similar sequence to				
						α	β	γ	δ	cryspo
Choiromyces venosus	RPA93728.1	DNA/RNA polymerase	502	fungi	Tuberaceae	√	√			
Dissophora globulifera	KAG0306265.1	hypothetical protein BGZ98_002688	687	fungi	Mortierellaceae				√	
Entomortierella beljakovae	KAF9427523.1	hypothetical protein BGZ76_002315	679	fungi	Mortierellaceae				√	
Haplosporangium bisporale	KAF8987572.1	hypothetical protein BGZ52_003881	529	fungi	Mortierellaceae			√		
Lunasporangiospora selenospora	KAF9584459.1	hypothetical protein BGW38_006380	603	fungi	Mortierellaceae				√	
Mortierella alpina	KAF9576628.1	hypothetical protein EC968_006975	717	fungi	Mortierellaceae				√	
Podila horticola	KAF9321465.1	hypothetical protein BG003_001587	578	fungi	Mortierellaceae			√		
Podila humilis	KAI9235092.1	hypothetical protein BYD32DRAFT_421964	576	fungi	Mortierellaceae			√		
Podila minutissima	KAG0359086.1	hypothetical protein BG005_001394	546	fungi	Mortierellaceae			√		
Podila verticillata	KAF9390496.1	hypothetical protein CPB97_009088	576	fungi	Mortierellaceae			√		
Amphibalanus amphitrite	KAF0302415.1	RNA-directed RNA polymerase	266	insect	Balanidae	√		√	√	
Anopheles sinensis	KFB47171.1	RNA-dependent RNA polymerase-like protein	308	insect	Culicidae			√		
Aphis glycines	KAE9528574.1	hypothetical protein AGLY_012149	315	insect	Aphididae				√	
Aquatica leii	KAK4887477.1	hypothetical protein RN001_003748	437	insect	Lampyridae			√	√	√
Arctia plantaginis	CAB3244939.1	unnamed protein product	354	insect	Erebidae			√		
Bemisia tabaci	CAH0389282.1	unnamed protein product	433	insect	Aleyrodidae			√	√	√
Bemisia tabaci	CAH0385445.1	unnamed protein product	254	insect	Aleyrodidae				√	
Bemisia tabaci	CAH0392084.1	unnamed protein product	361	insect	Aleyrodidae			√	√	√

Blattella germanica	PSN38948.1	hypothetical protein C0J52_08930	272	insect	Blattellidae			√	√	√
Bombus huntii	XP_050476991.1	uncharacterized protein LOC126866972	315	insect	Apidae	√		√		√
Ceutorhynchus assimilis	CAG9765738.1	unnamed protein product	364	insect	Curculionidae	√				
Chilo suppressalis	CAH0402448.1	unnamed protein product	385	insect	Crambidae		√	√	√	
Coccinella septempunctata	XP_044747513.1	uncharacterized protein LOC123308761	426	insect	Coccinellidae			√	√	√
Colletes gigas	XP_043262145.1	uncharacterized protein LOC122402968	388	insect	Colletidae			√	√	
Dinoponera quadriceps	XP_014468760.1	uncharacterized protein LOC106741364	391	insect	Formicidae			√	√	
Drosophila grimshawi	XP_032594741.1	uncharacterized protein LOC116805640	386	insect	Drosophilidae			√		
Homalodisca vitripennis	KAG8296771.1	hypothetical protein J6590_049796	512	insect	Cicadellidae		√	√	√	
Leptinotarsa decemlineata	XP_023013262.1	uncharacterized protein LOC111503242	316	insect	Chrysomelidae			√	√	√
Lutzomyia longipalpis	XP_055684456.1	uncharacterized protein LOC129790773	351	insect	Psychodidae			√		
Mischocyttarus mexicanus	KAI4493280.1	hypothetical protein M0802_009448	539	insect	Vespidae	√		√		
Odynerus spinipes	KAK2579121.1	hypothetical protein KPH14_001292	111	insect	Vespidae			√	√	√
Periplaneta americana	KAJ4448885.1	hypothetical protein ANN_00276	435	insect	Blattidae			√	√	
Phlebotomus papatasi	XP_055704333.1	uncharacterized protein LOC129802484	574	insect	Psychodidae			√	√	
Polistes exclamans	KAI4491421.1	hypothetical protein M0804_002813	538	insect	Vespidae	√		√	√	√
Pomphorhynchus laevis	KAI0983434.1	hypothetical protein GJ496_007148	347	insect	Pomphorhynchidae			√	√	√
Rhagoletis zephyria	XP_017484547.1	uncharacterized protein LOC108373198	248	insect	Tephritidae			√	√	√
Tenebrio molitor	KAJ3617823.1	hypothetical protein MTP99_006913	439	insect	Tenebrionidae			√		
Trichogramma pretiosum	XP_014230682.1	uncharacterized protein LOC106655011	397	insect	Trichogrammatidae			√	√	√
Wasmannia auropunctata	XP_011706462.1	uncharacterized protein LOC105461650	561	insect	Formicidae	√	√	√	√	√

Cylicostephanus goldi	VDN30519.1	unnamed protein product	114	nematode	Strongylidae		√	√		
Toxocara canis	KHN74396.1	RNA-directed RNA polymerase	452	nematode	Toxocaridae			√	√	√
Amborella trichopoda	ERN03490.1	hypothetical protein AMTR_s00003p00269640	314	plant	Amborellaceae				√	
Capsicum chinense	PHT96187.1	hypothetical protein BC332_34887	193	plant	Solanaceae				√	
Lolium perenne	AFA36554.1	putative RNA dependent RNA polymerase	182	plant	Poaceae				√	
Nymphaea thermarum	KAF3782951.1	hypothetical protein EJ110_NYTH20265	443	plant	Nymphaeaceae			√	√	
Picea sitchensis	ADE76327.1	unknown	413	plant	Pinaceae			√	√	√
Pyrus pyrifolia	BAA34783.1	RNA-dependent RNA polymerase	477	plant	Rosaceae			√	√	√
Rosa rugosa	XP_062028957.1	uncharacterized protein LOC133744954	507	plant	Rosaceae			√	√	
Tanacetum cinerariifolium	GEU95306.1	hypothetical protein	187	plant	Asteraceae				√	
Tanacetum cinerariifolium	GEZ38124.1	hypothetical protein	310	plant	Asteraceae	√	√	√		
Tanacetum cinerariifolium	GEZ39467.1	hypothetical protein	489	plant	Asteraceae	√	√	√		
Eimeria necatrix	XP_013435739.1	hypothetical protein ENH_00033460	214	protozoan	Eimeriidae				√	√
