# Supplementary material for: A Hypovirulence-Associated Partitivirus and Re-Examination of Horizontal Gene Transfer Between Partitiviruses and Cellular Organisms
Source: Int J Mol Sci. 2025 Apr 18;26(8):3853. doi: 10.3390/ijms26083853 (PMC12027680; doi:10.3390/ijms26083853)
Supplement: Supplementary file 1 [file ijms-26-03853-s001.zip › Table S4-20241006.pdf]

Table S4: Partitivirus CP-like proteins in cellular organisms

Scientific Name	Accession	protein	Length	Group	Family	Similar sequence to				
						α	β	γ	δ	cryspo
Asgard group archaeon	MCP8716971.1	hypothetical protein	306	archaea	Archaea					√
Podosphaera aphanis	KAI1001190.1	hypothetical protein K3495_g7013	299	fungi	Erysiphaceae		√			
Candida albicans	KAF6071127.1	hypothetical protein FOB64_001538	359	fungi	Debaryomycetaceae					√
Candida dubliniensis	XP_002419563.1	conserved hypothetical protein	359	fungi	Debaryomycetaceae					√
Candida maltosa	EMG45456.1	hypothetical protein G210_4360	340	fungi	Debaryomycetaceae					√
Candida maltosa	EMG45457.1	hypothetical protein G210_4361	256	fungi	Debaryomycetaceae					√
Candida tropicalis	XP_002551399.1	conserved hypothetical protein	272	fungi	Debaryomycetaceae					√
Candida tropicalis	XP_002551400.1	conserved hypothetical protein	306	fungi	Debaryomycetaceae					√
Candida viswanathii	RCK64498.1	capsid protein	313	fungi	Debaryomycetaceae					√
Kazachstania humilis	GMM55084.1	hypothetical protein DAKH74_017000	270	fungi	Saccharomycetaceae					√
Spathaspora passalidarum	XP_007376554.1	uncharacterized protein SPAPADRAFT_62370	305	fungi	Debaryomycetaceae					√
Diabrotica virgifera virgifera	XP_028152101.1	IAA-leucine resistant 2-like	562	insect	Chrysomelidae					
Frankliniella occidentalis	XP_026286351.1	IAA-leucine resistant 2-like	358	insect	Thripidae					
Phaedon cochleariae	CAH1171203.1	unnamed protein product	482	insect	Chrysomelidae	√				
Aphis craccivora	KAF0690353.1	Uncharacterized protein FWK35_00035662	355	insect	Aphididae		√			

Apolygus lucorum	KAF6207350.1	hypothetical protein GE061_018591	1114	insect	Miridae		√			
Contarinia nasturtii	XP_031639808.1	uncharacterized protein LOC116351805	428	insect	Cecidomyiidae		√			
Diabrotica balteata	CAG9840808.1	unnamed protein product	696	insect	Chrysomelidae		√			
Eretmocerus hayati	KAJ8682370.1	hypothetical protein QAD02_018162	269	insect	Aphelinidae		√			
Frankliniella fusca	KAK3928055.1	hypothetical protein KUF71_016338	677	insect	Thripidae		√			
Gonioctena quinquepunctata	KAG5871096.1	hypothetical protein JTB14_035793	435	insect	Chrysomelidae		√			
Ladona fulva	KAG8227153.1	hypothetical protein J437_LFUL001697	350	insect	Libellulidae		√			
Macrosiphum euphorbiae	CAI6353141.1	unnamed protein product	364	insect	Aphididae		√			
Pomphorhynchus laevis	KAI0983758.1	hypothetical protein GJ496_003929	505	insect	Pomphorhynchidae		√			
Psylliodes chrysocephala	CAH1114828.1	unnamed protein product	590	insect	Chrysomelidae		√			
Schistocerca gregaria	XP_049861534.1	uncharacterized protein LOC126355288	416	insect	Acrididae		√			
Trifolium medium	MCI33606.1	coat protein	141	plant	Fabaceae		√			
Blomia tropicalis	KAJ6218664.1	hypothetical protein RDWZM_009821	781	insect	Echimyopodidae			√		
Meloidogyne enterolobii	CAD2197219.1	unnamed protein product	451	nematode	Meloidogynidae			√		
Acer yangbiense	TXG73377.1	hypothetical protein EZV62_001956	320	plant	Aceraceae	√				
Arabidopsis thaliana	NP_683573.1	iaa-leucine resistant 2	386	plant	Brassicaceae	√				
Arabis alpina	KFK39178.1	hypothetical protein AALP_AA3G210400	383	plant	Brassicaceae	√				
Arabis nemorensis	VVA98099.1	unnamed protein product	376	plant	Brassicaceae	√				
Artemisia annua	PWA50825.1	hypothetical protein CTI12_AA469230	433	plant	Asteraceae	√				
Capsella rubella	XP_006300065.1	IAA-leucine resistant 2	417	plant	Brassicaceae					

Chenopodium quinoa	XP_021748749.1	IAA-leucine resistant 2-like	324	plant	Chenopodiaceae					
Coptis chinensis	KAF9589199.1	hypothetical protein IFM89_019708	302	plant	Coptidoideae	√				
Cuscuta epithymum	CAH9097587.1	unnamed protein product	411	plant	Convolvulaceae	√				
Cuscuta europaea	CAH9077133.1	unnamed protein product	411	plant	Convolvulaceae	√				
Erigeron canadensis	XP_043633383.1	IAA-leucine resistant 2-like	336	plant	Asteraceae	√				
Erythranthe guttata	EYU25902.1	hypothetical protein MIMGU	432	plant	Phrymaceae	√				
Eutrema salsugineum	XP_024013625.1	IAA-leucine resistant 2	406	plant	Umbelliferae					
Helianthus annuus	XP_022025252.2	IAA-leucine resistant 2	376	plant	Asteraceae	√				
Mercurialis annua	XP_050224102.1	IAA-leucine resistant 2	419	plant	Euphorbiaceae	√				
Nicotiana attenuata	OIT27845.1	hypothetical protein A4A49_22659	418	plant	Solanaceae	√				
Nicotiana sylvestris	XP_009801524.1	uncharacterized protein LOC104247267 isoform X1	236	plant	Solanaceae	√				
Papaver somniferum	XP_026379755.1	IAA-leucine resistant 2	382	plant	Papaveraceae					
Phtheirospermum japonicum	GFP80994.1	capsid protein	386	plant	Orobanchaceae	√				
Smallanthus sonchifolius	KAI3821176.1	hypothetical protein L1987_08735	355	plant	Asteraceae	√				
Spinacia oleracea	XP_021850321.1	IAA-leucine resistant 2	419	plant	Chenopodiaceae					
Trifolium medium	MCI04234.1	protein IAA-LEUCINE RESISTANT 2	272	plant	Fabaceae	√				
Trifolium subterraneum	GAU42386.1	hypothetical protein TSUD_123820	390	plant	Fabaceae	√				
Acer saccharum	KAK1554320.1	hypothetical protein Q3G72_010652	222	plant	Aceraceae				√	
Acer yangbiense	TXG56362.1	hypothetical protein EZV62_017675	260	plant	Aceraceae				√	
Arabidopsis arenosa	CAE6139657.1	unnamed protein product	1015	plant	Brassicaceae				√	

Arabidopsis lyrata	CAH8273366.1	unnamed protein product	205	plant	Brassicaceae				√	
Arabidopsis suecica	KAG7557834.1	F-box-like domain superfamily	649	plant	Brassicaceae				√	
Arabidopsis thaliana	NP_001031631.1	capsid-like protein	144	plant	Brassicaceae				√	
Artemisia annua	PWA59773.1	coat protein	359	plant	Asteraceae				√	
Asparagus officinalis	XP_020261848.1	uncharacterized protein LOC109837880 isoform X2	618	plant	Asparagaceae				√	
Bauhinia variegata	KAI4322747.1	hypothetical protein L6164_022413	266	plant	Fabaceae				√	
Cannabis sativa	KAF4388417.1	hypothetical protein G4B88_013254	351	plant	Cannabaceae				√	
Capsicum baccatum	PHT37451.1	hypothetical protein CQW23_21024	180	plant	Solanaceae				√	
Cephalotus follicularis	GAV63346.1	hypothetical protein CFOL_v3_06864	338	plant	Cephalotaceae				√	
Cichorium endivia	KAI3519653.1	hypothetical protein L1887_08868	325	plant	Asteraceae				√	
Cichorium intybus	KAI3698860.1	hypothetical protein L2E82_42735	350	plant	Asteraceae				√	
Corchorus olitorius	OMO82839.1	hypothetical protein COLO4_22802	292	plant	Malvaceae				√	
Cucumis melo	XP_008459635.1	uncharacterized protein LOC103498698	210	plant	Cucurbitaceae				√	
Cuscuta campestris	VFQ64431.1	unnamed protein product	197	plant	Convolvulaceae				√	
Dendrobium chrysotoxum	KAH0457705.1	hypothetical protein IEQ34_013020	624	plant	Orchidaceae				√	
Dorcoceras hygrometricum	KZV15179.1	hypothetical protein F511_33665	266	plant	Gesneriaceae				√	
Escallonia herrerae	KAK3041664.1	hypothetical protein RJ639_001474	228	plant	Escalloniaceae				√	
Escallonia rubra	KAK2985298.1	hypothetical protein RJ640_024294	305	plant	Escalloniaceae				√	
Euphorbia peplus	WCJ29078.1	hypothetical protein M5689_010737	313	plant	Euphorbiaceae				√	
Ficus carica	GMN55363.1	hypothetical protein TIFTF001_024483	271	plant	Moraceae				√	

Fragaria vesca	XP_011464272.1	PREDICTED: uncharacterized protein LOC105351507	380	plant	Rosaceae				√	
Glycine max	KAH1199504.1	hypothetical protein GmHk_18G052849	101	plant	Fabaceae				√	
Glycine soja	KAG4925373.1	hypothetical protein JHK87_050913	122	plant	Fabaceae				√	
Gossypium anomalum	KAG8471443.1	hypothetical protein CXB51_036179	844	plant	Malvaceae				√	
Gossypium arboreum	XP_017644567.2	uncharacterized protein LOC108485246 isoform X1	466	plant	Malvaceae				√	
Gossypium australe	KAA3485722.1	ABC transporter F family member 1	233	plant	Malvaceae				√	
Gossypium barbadense	KAB2050559.1	hypothetical protein ES319_A13G254600v1	472	plant	Malvaceae				√	
Gossypium darwinii	TYG88125.1	hypothetical protein ES288_A13G270000v1	472	plant	Malvaceae				√	
Gossypium harknessii	MBA0817279.1	hypothetical protein	251	plant	Malvaceae				√	
Gossypium hirsutum	XP_016738288.1	uncharacterized protein LOC107948300 isoform X1	219	plant	Malvaceae				√	
Gossypium lobatum	MBA0574659.1	hypothetical protein	335	plant	Malvaceae				√	
Gossypium mustelinum	TYJ02983.1	hypothetical protein E1A91_A13G267000v1	447	plant	Malvaceae				√	
Gossypium raimondii	KJB83871.1	hypothetical protein B456_013G268900	313	plant	Malvaceae				√	
Gossypium stocksii	KAH1033784.1	hypothetical protein J1N35_045958	447	plant	Malvaceae				√	
Gossypium tomentosum	TYH93772.1	hypothetical protein ES332_A13G276600v1	286	plant	Malvaceae				√	
Helianthus annuus	KAF5764356.1	hypothetical protein HanXRQr2_Chr15g0691081	373	plant	Asteraceae				√	
Hibiscus syriacus	KAE8660593.1	hypothetical protein F3Y22_tig00116951pilonHSYRG00466	377	plant	Malvaceae				√	
Hibiscus trionum	GMI96611.1	hypothetical protein HRI_003330400	738	plant	Malvaceae				√	
Ipomoea trifida	GLL40948.1	crossover junction endonuclease MUS81 isoform X4	897	plant	Convolvulaceae				√	
Lactuca saligna	CAI9275747.1	unnamed protein product	332	plant	Asteraceae				√	

Lactuca virosa	CAH1414356.1	unnamed protein product	345	plant	Asteraceae				√	
Lolium multiflorum	KAK1614176.1	hypothetical protein QYE76_019693	474	plant	Poaceae				√	
Lolium rigidum	XP_047080872.1	uncharacterized protein LOC124691628	713	plant	Poaceae				√	
Malus baccata	TQD85618.1	hypothetical protein C1H46_028792	399	plant	Rosaceae				√	
Manihot esculenta	XP_021620377.1	uncharacterized protein LOC110620805	339	plant	Euphorbiaceae				√	
Morus notabilis	XP_024027537.1	uncharacterized protein LOC21397379 isoform X2	320	plant	Moraceae				√	
Nicotiana attenuata	OIT06636.1	hypothetical protein A4A49_02124	263	plant	Solanaceae				√	
Nyssa sinensis	KAA8548732.1	hypothetical protein F0562_000416	100	plant	Nyssaceae				√	
Papaver armeniacum	KAI3944093.1	hypothetical protein MKW92_037295	190	plant	Papaveraceae				√	
Papaver atlanticum	KAI3959368.1	hypothetical protein MKW98_018958	452	plant	Papaveraceae				√	
Papaver bracteatum	KAI3849250.1	hypothetical protein MKX03_001649	141	plant	Papaveraceae				√	
Papaver somniferum	RZC49540.1	hypothetical protein C5167_017961	296	plant	Papaveraceae				√	
Paspalum vaginatum	KAJ1282549.1	hypothetical protein BS78_03G061000	202	plant	Poaceae				√	
Phtheirospermum japonicum	GFP96432.1	hypothetical protein PHJA_001787300	206	plant	Orobanchaceae				√	
Picea sitchensis	ABR17456.1	unknown protein	195	plant	Pinaceae				√	
Sesbania bispinosa	KAJ1422759.1	hypothetical protein SESBI_12805	215	plant	Fabaceae				√	
Smallanthus sonchifolius	KAI3717401.1	hypothetical protein L1987_68991	338	plant	Asteraceae				√	
Striga asiatica	GER31701.1	protein kinase	881	plant	Orobanchaceae				√	
Striga hermonthica	CAA0835223.1	Unknown protein	118	plant	Orobanchaceae				√	
Tagetes erecta	KAK1422994.1	hypothetical protein QVD17_18287	373	plant	Asteraceae				√	

Thalictrum thalictroides	KAF5182510.1	coat protein	195	plant	Ranunculaceae				√	
Trifolium pratense	PNX60680.1	hypothetical protein L195_g052054	300	plant	Fabaceae				√	
Vigna angularis	KOM46435.1	hypothetical protein LR48_Vigan07g013900	345	plant	Fabaceae				√	
Vigna radiata	XP_022635923.1	uncharacterized protein LOC111241564	190	plant	Fabaceae				√	
Vigna umbellata	XP_047170224.1	uncharacterized protein LOC124838616	368	plant	Fabaceae				√	
Vigna unguiculata	QCD86406.1	hypothetical protein DEO72_LG3g928	269	plant	Fabaceae				√	
Cryptosporidium felis	ABB02502.1	uncharacterized protein J8A68_000753	314	protozoan	Cryptosporidiidae					√
Cryptosporidium hominis	ABB02500.1	conserved hypothetical protein	362	protozoan	Cryptosporidiidae					√
Cryptosporidium meleagridis	ABB02501.1	hypothetical protein JA1_001406	307	protozoan	Cryptosporidiidae					√
