# Supplementary figures and images for: A Hypovirulence-Associated Partitivirus and Re-Examination of Horizontal Gene Transfer Between Partitiviruses and Cellular Organisms
Source: Int J Mol Sci. 2025 Apr 18;26(8):3853. doi: 10.3390/ijms26083853 (PMC12027680; doi:10.3390/ijms26083853)

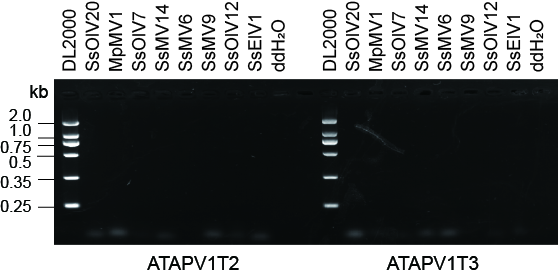

Supplement: Supplementary file 1 [file ijms-26-03853-s001.zip › Figure S1.tif]

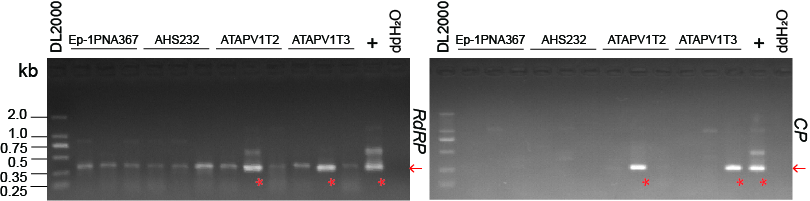

Supplement: Supplementary file 1 [file ijms-26-03853-s001.zip › Figure S2.tif]

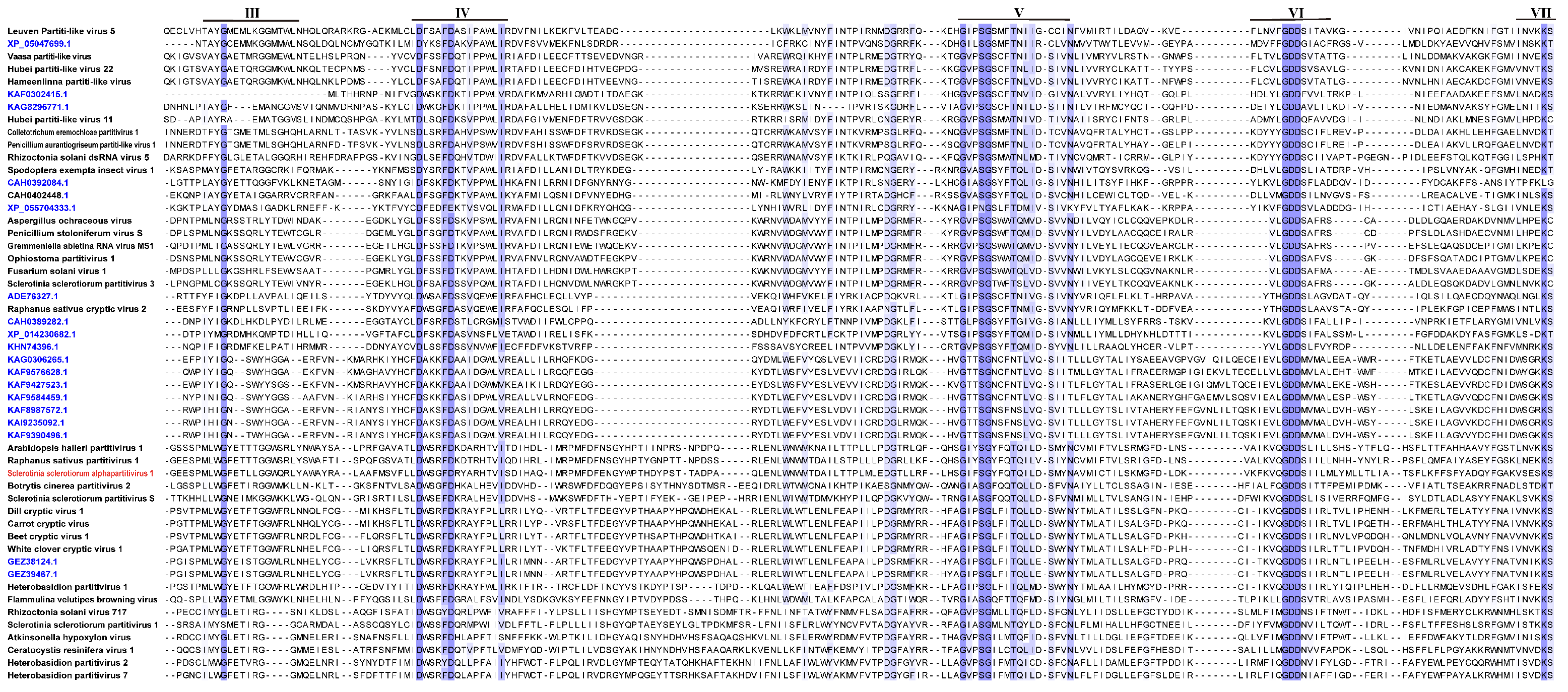

Supplement: Supplementary file 1 [file ijms-26-03853-s001.zip › Figure S3.tif]
